# Molecular Analyses of the Distribution and Function of Diazotrophic Rhizobia and Methanotrophs in the Tissues and Rhizosphere of Non-Leguminous Plants

**DOI:** 10.3390/plants8100408

**Published:** 2019-10-11

**Authors:** Tadakatsu Yoneyama, Junko Terakado-Tonooka, Zhihua Bao, Kiwamu Minamisawa

**Affiliations:** 1 Department of Applied Biological Chemistry, University of Tokyo, Yayoi 1-1-1, Bunkyo-ku, Tokyo 113-8657, Japan; 2 National Agriculture and Food Research Organization, Kannondai 3-1-1, Tsukuba, Ibaraki 305-8666, Japan; 3 School of Ecology and Environment, Inner Mongolia University, 235 West University Blvd., Hohhot 010021, Inner Mongolia, China; bao1016@163.com; 4 Graduate School of Life Sciences, Tohoku University, Katahira, Aoba-ku, Sendai, Miyagi 980-8577, Japan

**Keywords:** biological nitrogen fixation, endophytic diazotrophs, methanotrophs, non-leguminous plants, rhizobia

## Abstract

Biological nitrogen fixation (BNF) by plants and its bacterial associations represent an important natural system for capturing atmospheric dinitrogen (N_2_) and processing it into a reactive form of nitrogen through enzymatic reduction. The study of BNF in non-leguminous plants has been difficult compared to nodule-localized BNF in leguminous plants because of the diverse sites of N_2_ fixation in non-leguminous plants. Identification of the involved N_2_-fixing bacteria has also been difficult because the major nitrogen fixers were often lost during isolation attempts. The past 20 years of molecular analyses has led to the identification of N_2_ fixation sites and active nitrogen fixers in tissues and the rhizosphere of non-leguminous plants. Here, we examined BNF hotspots in six reported non-leguminous plants. Novel rhizobia and methanotrophs were found to be abundantly present in the free-living state at sites where carbon and energy sources were predominantly available. In the carbon-rich apoplasts of plant tissues, rhizobia such as *Bradyrhizobium* spp. microaerobically fix N_2_. In paddy rice fields, methane molecules generated under anoxia are oxidized by xylem aerenchyma-transported oxygen with the simultaneous fixation of N_2_ by methane-oxidizing methanotrophs. We discuss the effective functions of the rhizobia and methanotrophs in non-legumes for the acquisition of fixed nitrogen in addition to research perspectives.

## 1. Introduction

**Figure 1:**
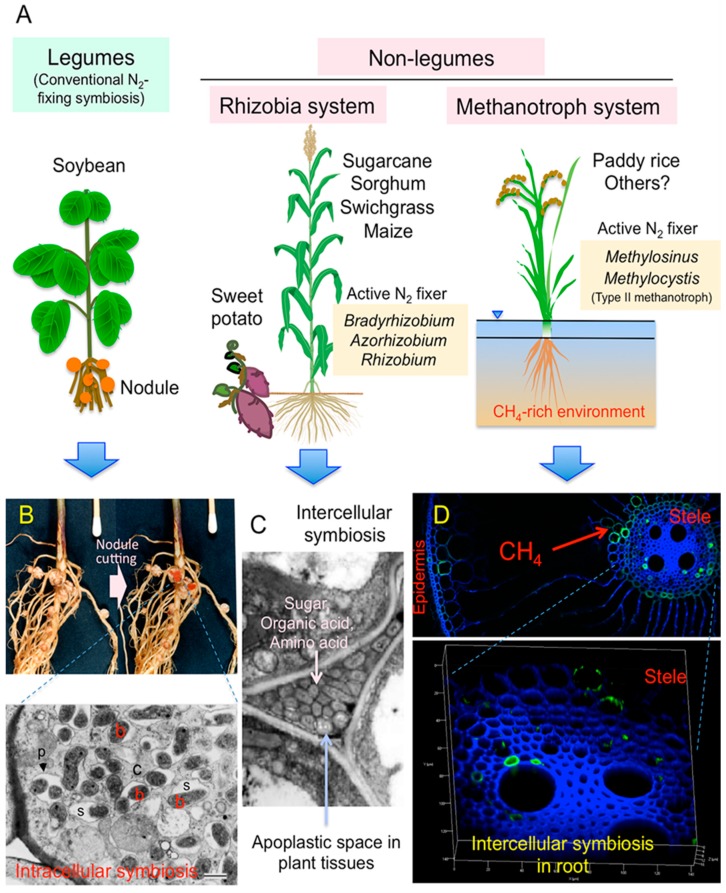
Comparison of N_2_-fixing bacteria associated with legume and non-legume. (**A**) *Bradyrhizobium*, *Azorhizobium*, and *Rhizobium* were N_2_ fixers in non-legume tissues of sugarcane, sorghum, switchgrass, and sweet potato (Tables 2–6), which were termed the “Rhizobia system” [2] On the other hand, *Methylosinus* and *Methylocystis* were found as active N_2_ fixers in paddy rice roots under methane-rich environments (Table 7), and were termed the “Methanotroph system” [2]. Note that the “Rhizobia system” and the “Methanotroph system” involved intercellular symbiosis that was quite different from the “Rhizosphere system” [2]. (**B**) Root nodules of soybean (*Glycine max*. cv. Enrei) and TEM(transmission electron microscope) micrograph of infected cells in the nodule. “b”, “c”, “p” and “s” indicates bacteroids of *Bradyrhizobium diazoefficiens*, cytoplasm of plant cell, peribacteriodal membrane, and symbiosome space, respectively. The nodules show typical intracellular symbiosis for nitrogen fixation. (**C**) TEM photograph showing colonization N_2_-fixing *Herbaspirillum* sp. in the intercellular spaces of wild rice tissue [3]. This is a conceptional image of intercellular symbiosis between non-legumes and *Rhizobium/Bradyrhizobium* because these combinations have not yet observed by TEM. (**D**) Catalyzed reporter deposition-fluorescence in situ hybridization (CARD-FISH) detection of Methylocystaceae members (type II methanotrophs including *Methylosinus* and *Methylocystis*) in roots of field-grown rice (*Oryza sativa* Nipponbare) by confocal laser scanning microscopy [4]. The Alexa Fluor 488 fluorescence of the Ma450 probe for Methylocystaceae members is shown in green, whereas the autofluorescence of cell wall of rice root is blue [4]). A greenish signal corresponding to type II methanotrophs was detected in the intercellular spaces around the stele of root tissue of paddy rice [4].

Biological nitrogen fixation (BNF) by plant–rhizobia symbiotic systems is mediated by a specialized plant organ known as a nodule (Figure 1A,B). The nodules use atmospheric nitrogen (N_2_)—a huge pool of inert gas (80% of the atmosphere)—as a nitrogen source [1]. The pathway for the biological reduction of inert N_2_ into the reactive compound NH_3_ (ammonia) under microaerobic conditions is as follows:

N_2_ + 8H^+^ + 8e^−^ + 16Mg-ATP → 2NH_3_ + H_2_ + 16Mg-ADP + 16 Pi

In the nodules of leguminous plants, a combination of the two proteins, nitrogenase reductase and dinitrogenase, reduces N_2_ to NH_3_ under low redox conditions (−500 mV) using a large amount of reductant nicotinamide adenine dinucleotide (NADH) and energy adenosine triphosphate (ATP), which are synthesized from supplied photosynthates. The fixed nitrogen (NH_3_) is transferred from the nodule’s bacteroids to the host plant’s cells through the symbiosome space as ammonium/amino acids for further assimilation and export to the host plant (Figure 1B).

In a nonsymbiotic system of associative N_2_ fixation, which commonly occurs in the rhizosphere of higher plants, N_2_-fixing bacteria fix N_2_ by using carbon and energy sources supplied from the environment [5]. The bacteria release the fixed N, probably following cell lysis [6]. In the rhizosphere-associated system, diazotrophic bacteria such as *Beijerinckia, Azotobacter,* and *Klebsiella* fix N_2_ in the free-living state using carbohydrates from the soil environment, which are byproducts of root excretion and the degradation of soil organic matter. However, diverse and numerous N_2_-fixing and non-fixing bacteria residing in the rhizosphere may compete for these carbohydrates [2]. Such competitive consumption of carbon compounds by rhizosphere bacteria results in a low level of N_2_ fixation.

In our previous review of bacterial N_2_-fixation systems in association with soil-grown sugarcane (*Saccharum* spp.), sweet potato (*Ipomoea batatas* L.), and paddy rice (*Oryza sativa* L.) [2], several lines of evidence demonstrated that the magnitudes of associative (nonsymbiotic) N_2_ fixation in sugarcane [7], sweet potato [8], and paddy rice [9] were larger than those of commonly occurring rhizosphere N_2_ fixation. Active expression of the dinitrogenase reductase-encoding gene (*nifH*) in phylogenetically similar *Bradyrhizobium* spp. and *Azorhizobium* sp. was observed in the N_2_-fixing sugarcane stems and sweet potato stems and tubers, called the “rhizobia system” (Figure 1C). These rhizobia microaerobically fix N_2_ in the carbon compound-rich apoplasts. *Gluconacetobacter diazotrophicus* and *Herbaspirillum* spp. were previously isolated from inside sugarcane and many other plants as candidates of endophytic N_2_ fixers, but molecular analyses have suggested that these bacteria may function as the producers of phytohormonal substances, but not as active N_2_ fixers. In paddy rice fields, methane is produced from organic compounds in anoxia and oxidized by contacting oxygen gas. Active N_2_ fixation by methane-oxidizing methanotrophs such as *Methylosinus* sp. takes place in the root tissues (aerenchyma) as well as in the surface soil, called the “methanotroph system” (Figure 1A,D).

Here, we describe the culture-independent molecular analyses that were conducted over the period of the last 20 years to identify the association between non-leguminous plants and N_2_-fixing bacteria in the plant tissues and rhizosphere. In Section 2, two case studies describe the detection of *nifH* gene expression (mRNA) in N_2_-fixing sugarcane stem and the characterization of small quantities of proteins for nitrogen fixation and methane oxidation using an enrichment technique for bacterial cells residing in plant tissues. In Section 3, molecular analyses were conducted for the presence and expression of *nif* genes and proteomics analyses for nitrogenase and methane oxidizing proteins in six plant species. In Section 4 and Section 5, we examine the physiological and ecological features of the rhizobia and methanotroph systems across plant species. Finally, in Section 6, we discuss perspectives for the future research of rhizobia and methanotrophs in non-legumes.

## 2. Case Studies to Search the Actively N_2_-Fixing Diazotrophs in Non-Legumes

### 2.1. Detection of Expression of nifH Genes in Young Sugarcane Stems

The detection of *nifH* DNA in plant tissues indicates the presence/distribution of diazotrophs and the expression of *nifH* genes indicates the possibility of active N_2_ fixation by diazotrophs. In the soft stem tissues of N_2_-fixing (studied using dilution of fertilizer containing ^15^N isotopes) sugarcane, the expression of *nifH* genes was detected based on RT-PCR, whereas the non-N_2_-fixing stem tissues did not show any evidence of *nifH* expression [10]. The extraction of *nifH* RNA from mature hard stem tissues was not successful, probably due to low extractivity and/or low quantities of *nifH* RNA.

RNA isolation and reverse transcription into cDNA was conducted as previously reported [10]. Fresh sugarcane tissues were ground in liquid nitrogen as soon as possible after harvest for RNA extraction and purification. DNA contaminants were eliminated and RNA was transcribed. The 16S rDNA regions were amplified using RT-PCR and the primers 968F—AAC GCG AAG AAC CTT AC and 1401R—CGG TGT GTA CAA GAC CC to evaluate the quality of the RNA [10].

PCR amplification, cloning, and sequencing of *nifH* gene segments were conducted as previously reported [10]. Using the RT-PCR product as a template, the *nifH* fragments were amplified by nested PCR with *Taq* DNA polymerase: IGK3 and VCG primers were used in the first round followed by KAD3 and DVV primers for the second round of amplification [11]. The PCR conditions were as follows: one cycle at 94 °C for 2 min; 30 cycles at 94 °C for 30 s, 50 °C for 1 min, 72 °C for 1 min; and one cycle at 72 °C for 3 min. The *nifH* gene fragment of 310 bp was cloned into *Escherichia coli*. Recombinant colonies were screened on Luria-Bertani agar plates containing 50 µg mL^−1^ of ampicillin, and colony PCR with T7 and T3 primers was performed. The PCR products were cleaned up using ExoSAP-IT. Sequences were determined using an ABI 3100 genetic analyzer.

Recently, the efficiency of the *nifH* PCR primer sets were examined for those from five laboratories [12] and the primer set called the Ando primer set, which was described above and has been previously reported [11], with a modified annealing temperature of 58 °C was recommended as this approach captured the largest diversity of *nifH* templates. 

### 2.2. Detection of Diazotrophic Methanotrophs in Rice Roots by Metaproteomics

To obtain the bacterial cell-enriched fraction, the bacterial cells were extracted from homogenized root tissues (approximately 100 g) of the paddy field-grown rice plants by a series of different centrifugation steps followed by a density gradient centrifugation [13]. Proteins were then extracted from the bacterial cells [4]. The abundances of peptides from the proteins involved in methane oxidation (particulate/soluble methane monooxygenase (pMMO/sMMO), methanol dehydrogenase (MxaFI), formaldehyde dehydrogenase (FAD), formate dehydrogenase (FDH)) and N_2_ fixation (NifH, NifD, NifK, VnfD) in the rice root were determined by metaproteomic analysis based on metagenome analysis [4]. 

An aliquot of the proteins (50 μg) was separated by 12.5% SDS-PAGE and stained with Coomassie blue. The gel lanes were cut into 60 strips, each approximately 1 mm wide. The gel strips were completely destained with 30% acetonitrile–25 mM NH_4_HCO_3_ mixture, reduced with 10 mM dithiothreitol, and alkylated with 55 mM iodoacetamide. After the gel strips were completely dried, they were digested with 40 μL of sequencing-grade modified trypsin (12.5 ng/μL trypsin in 50 mM NH_4_HCO_3_) by incubation at 37 °C overnight [14].

Nanoliquid chromatography (LC)–electrospray ionization–tandem mass spectrometry (MS/MS) analysis was performed for the peptide mixtures using an LTQ ion-trap MS coupled with a multidimensional high-performance LC Paradigm MS2 chromatograph and a nanospray electrospray ionization device. The tryptic peptide spectra were recorded in an *m/z* range of 450–1800 [14]. The obtained MS/MS data were searched against the rice root microbiome database that was constructed using metagenome data targeting the same rice root samples [4]

## 3. Molecular Analyses of Diazotrophs in Non-Legumes 

### 3.1. Endophytic Diazotrophs in Maize Plants

N_2_-fixing (*nifH* holding) endophytic *Burkholderia* spp. were isolated from the shoot and root tissues of 30-day-old maize (*Zea mays* L.) seedlings that were grown in soil rich in Mexican *Burkholderia* [15,16]. Inoculation of maize with *Burkholderia* isolates induced their dense colonization in the maize tissues.

The biodiversity of the diazotrophic bacteria present in the stem, root, and rhizosphere soil collected from six maize grown regions in Rio Grande do Sul, Brazil, was assessed by *nifH* DNA sequencing [17]. The following N_2_-fixing bacteria were found to be abundant as deduced from the detection of their *nifH* genes: *Ideonella*, *Azospirillum*, *Klebsiella*, *Herbaspirillum*, and *Raoultella* in the stem; *Bradyrhizobium*, *Azospirillum*, and *Klebsiella* in the root; and *Bradyrhizobium*, *Ideonella*, *Azospirillum*, and *Klebsiella* in the rhizosphere soil (Table 1). Other N_2_-fixing bacteria found in the rhizosphere soil were from diverse genera such as *Methylocystis*, *Beijerinckia*, *Geobacter*, *Rhodovulum*, *Methylobacterium*, *Gluconacetobacter*, *Methylocella*, and *Delftia*, while those in the stem and root corresponded to less diverse genera such as *Methylosinus, Rhizobium,* and *Dechloromonas*.

Although the diazotrophic bacteria were not identified, a landrace of maize in Sierra Mixe, Mexico developed carbohydrate-rich mucilage in their root fixed N_2_, accounting for as much as 29–82% of nitrogen nutrition in the nitrogen-depleted soils [18]. The *nifD* genes of the microbiome were abundantly extracted from the stem, mucilage, and rhizosphere of the Sierra Mixe maize. 

### 3.2. Endophytic Diazotrophs in Sorghum

The search for *nifH* DNA in the rhizosphere of two cultivars of sorghum (*Sorghum bicolor*) grown with low and high doses of nitrogen fertilizer in a Cerrado soil of Brazil [19] indicated a high abundance of *Bradyrhizobium* spp. such as *B.* sp. AF48469, followed by *Azohydromonas australica* and *Ideonella* sp. (Table 2). The *nifH* clones derived from *Delftia tsuruhatensis* were found in the rhizosphere of both cultivars with high-nitrogen fertilizer, while those from *Methylocystis* sp. were detected only in plants with low-nitrogen fertilizer.

The activities of ^15^N_2_ fixation of the roots harvested from sorghum lines KM1 and KM2 were highest at the late growth stage [20]. The N_2_-fixing bacterial cells extracted from the roots were subject to metagenomic analysis for *nifHDK* genes and proteomic analysis of corresponding peptides. The N_2_-fixing bacteria present in the roots were predominantly deduced to be the *Bradyrhizobium* species including non-nodulating *Bradyrhizobium* sp. S23321 and photosynthetic *B. oligotrophicum* S58^T^, and a small percentage was derived from *Azorhizobium* sp. (Table 2). The high abundance of the two *Bradyrhizobium* spp. were also validated by the proteomic analysis using *nifHDK*-derived peptides (Table 2), indicating that the bacteria expressed nitrogenase in the roots. It is noteworthy that the two *Bradyrhizobium* spp. isolated from the root tissues had N_2_-fixing activity under free-living conditions [20].

### 3.3. Endophytic Diazotrophs in Switchgrass

The first molecular analysis of diazotrophs in the shoot and root tissues of switchgrass (*Panicum virgatum* L.) was reported for the plant samples collected from the tallgrass prairie, Oklahoma, USA [21]. The DNA of the shoot and root tissues was analyzed and revealed the high abundance of *nifH* in photosynthetic *Bradyrhizobium* spp. including strains BTAi1 and MAFF210318, *Burkholderia* spp., *Sphingomonas azotifigens*, *Anaeromyxobacter* spp., *Geobacter* spp., and *Rhizobium helanshanense*. Distinct distributions of diazotrophs between the shoot and root were observed: *Azospirillum lipoferum*, *Klebsiella* sp., *Desulfuromonas* spp., and *Syntrophobacter fumaroxidans* in the shoot, and *Methylocystis* sp. and *Methylobacterium nodulans* in the root (Table 3).

The expression of *nifH* RNA in root tissues was characterized by RT-PCR. The data corresponding to *nifH* DNA and *nifH* cDNA from the root tissues showed that *nifH* was expressed in *Burkholderia* spp., *Methylobacterium nodulans*, *Rhizobium helanshanense*, and *Geobacter* sp., whereas *nifH* was not expressed in *Bradyrhizobium* spp., *Sphingomonas azotifigens*, and *Methylocystis* sp. in the root tissues (Table 3). A study for the function of *Burkholderia phytofirmans* and *Sphingomonas* sp. in switchgrass suggested that they promoted plant growth under limited nitrogen supply [22]. Another recent investigation on the stage of ^15^N_2_ fixation by switchgrass roots showed that the highest ^15^N fixation occurred after the plants’ senescence, suggesting that the carbon compounds were released during senescence [23].

### 3.4. Endophytic Diazotrophs in Sugarcane Plants

The endophytic diazotrophic bacteria from shoot extracts of sugarcane (*Saccharum* spp.) were isolated in Brazil by examining their N_2_-fixing (acetylene reduction) ability in N-free semisolid medium; first, *Acetobacter* (later reclassified into *Gluconacetobacter*) *diazotrophicus* [24,25] and, later, *Herbaspirillum rubrisubalbicans* and *H. seropedicae* [26,27]. The N_2_ fixation-related genes *nifA*, *nifB, nifHDK*, and *ntrBC* were identified in *G. diazotrophicus* [28,29]. *G. diazotrophicus* was also isolated from the roots and stems of sugarcane cultivars in Australia [30], México (at high-N fertilization, [31]), and Miyako Island, Japan [32].

*Herbaspirillum* spp. produces indole-3-acetic acid (IAA) and gibberellins (GAs) [33]. Another phytohormone, ethylene, may also be involved in the growth of sugarcane [34]. Some diazotrophic and IAA-producing *Paraburkholderia* spp. (*P. unamae, P. tropica*) have been found in field-grown Brazilian and Mexican sugarcanes [16,35]. Thus, regarding the roles of endophytic *Azospirillum* sp., *Gluconacetobacter* sp., *Herbaspirillum* sp., and *Paraburkholderia* sp., the production of phytohormonal substances such as IAA, cytokinins, and Gas, which induces the active proliferation of roots and active uptake of water and mineral, is very prominent, whereas the nitrogen-fixating activity is of a lesser significance than initially anticipated [36,37,38,39,40].

The search for sugarcane *nifH* DNA sequences and their closest genus (deduced by their similarity) was first reported for stems of mature sugarcane harvested in Miyako Island, Okinawa, and carried to Tsukuba, Ibaraki, Japan [11]. The stems of two cultivars (KF92-93, NCo310) contained *nifH* DNA mostly from *Bradyrhizobium* spp., whereas the stems of cv. NiF8 contained genes presumably from *Klebsiella* spp. and *Serratia* spp. (Table 4). The presence and expression of *nifH* was not detected in *Gluconacetobacter diazotrophicus*.

In order to extract *nifH* RNA shortly after harvest, young sugarcane plants grown from the cut stem from cultivar NiF8 (Miyako Island) were grown in soil pots in the greenhouse under high and low temperatures. Both *nifH* DNA and *nifH* RNA were detected in the stems under high temperature, whereas they were not present in the stems under low temperature. On the other hand, *nifH* DNA and *nifH* RNA were detected in the roots of both plants grown under high and low temperatures. 

The *nifH* DNA and *nifH* RNA from the stems of the plants grown under high temperature showed that they were largely derived from the bacteria having genes close to *Bradyrhizobium* spp. such as photosynthetic *B.* sp. BTAi1 and *B.* sp. IRBG230, and non-photosynthetic *Bradyrhizobium* sp. MAFF210318 and *Azorhizobium caulinodans* (Table 4). The root *nifH* DNA and *nifH* RNA were commonly derived from *Bradyrhizobium* spp. and *Azorhizobium caulinodans,* specifically, *Rhizobium daejonense* and *Beijerinckia derxii* in commercial soil; *Rhizobium daejonense*, *Methylocystis* sp. *Methylobacterium* sp., and *Burkholderia ferrariae* in Ishigaki soil; and *Methylobacterium nodulans*, *Methylocella silvestris*, and *Azonexus caeni* in Tanegashima soil (Table 4).

The assay for *nifH* RNA expression in Brazil sugarcane was conducted using mature plants. RNA was extracted from the leaf sheath and root of 6-month-old sugarcane plants [41] and also extracted from the root of 5-month-old plants [42]. The *nifH* RNA extraction from the leaf sheath and root indicated that they were derived from *Rhizobium* spp., *Paraburkholderia tropica*, and *Idenella/Herbaspirillum*-like bacteria (previously confirmed by isolation and culture method) in the leaf sheath and *Azospirillum brasilense*, *Bradyrhizobium* spp., *Methylocapsa* spp., *Paraburkholderia tropica*, and *Idenella/Herbaspirillum*-like bacteria in the roots (Table 5). 

Isolation of nodule-forming bacteria from the root extracts of trap plants (siratro, cowpea) showed that the *nifH* genes were largely derived from *Bradyrhizobium* spp. and *Bradyrhizobium sacchari* sp. nov. [43], which forms nodules on cowpea, *Cajanus cajan*, and siratro, but not on soybean. Importantly, the *Bradyrhizobium* sp. isolated and identified from the root extracts of Brazilian sugarcane expressed nitrogenase under free-living conditions [43]. Four of the six direct plate isolates were mostly *Bradyrhizobium* sp. and did not contain *nodC* genes. The other isolates of N_2_-fixing bacteria were *Rhizobium* sp., *Methylobacterium*, and *Herbaspirillum* (Table 5).

The stem and root tissues collected from Liberia farm, Columbia, contained *nifH* genes of free-living *Bradyrhizobium* sp. and other genera [12]. Australian commercial sugarcanes have not previously shown any significant fixed-N input using a natural ^15^N method [44]. A molecular study on N_2_-fixing bacterial communities concluded that the root-associated diazotrophs were very scant, and reduced application of N fertilizer did not cause any increase in the abundance of these diazotrophs [45]. A new species, *Burkholderia australis*, was isolated from Australian sugarcane root based on the *nifH* DNA search [46].

### 3.5. Endophytic Diazotrophs in Sweet Potato Plants

Reiter et al. [47] applied a culture-independent approach to estimate the *nifH* DNA-carrying diazotrophs in the stem and tuber samples harvested from African sweet potatoes (*Ipomoea balatas*) grown in Uganda and Kenya. About 50% of the identified sequences were derived from rhizobia such as *Sinorhizobium meliloti*, *Bradyrhizobium japonicum*, *Bradyrhizobium* sp., and *Rhizobium etli*; few sequences were derived from *Paenibacillus odorifer*, *Clostridium pasteurianum*, and *Azoarcus* sp. BH72 (Table 6). The *nifH* DNA was also isolated from the stem and tuber samples from several-months-old sweet potatoes grown at a Tsukuba field, where a high N_2_ fixation was recorded [48]. This study indicated that the *nifH* DNA sequences were mostly derived from rhizobia such as *Bradyrhizobium* sp. including strains MAFF210318 and IRBG230, *Azorhizobium caulinodans*, *Rhizobium leguminosarum, Sinorhizobium* sp., and *Herbaspirillum seropedicae, Paraburkholderia unamae*, *Azohydromonas* sp., *Pelomonas* sp., and *Bacillus* sp. BT95 sequences were detected at a lower frequency. However, no *nifH* DNA sequences were detected for *Gluconacetobacter diazotrophicus* [48]. 

The *nifH* gene sequences collected from the stem and tuber mRNA samples were mostly derived from *Bradyrhizobium* sp. and some sequences were derived from *Bacillus* sp. and *Pelomonas* sp., but no *nifH* mRNA sequences were detected for those of *Gluconacetobacter diazotrophicus*, *Klebsiella* sp., or *Herbaspirillum* sp. [48]

From surface-sterilized sweet potatoes, Terakado-Tonooka et al. [49] isolated an endophytic diazotroph, *Bradyrhizobium* sp. strain AT1, which had a *nifH* sequence similar (96%) to that of *Aeschynomene* stem-nodulating *Bradyrhizobium.* sp. ORS391, a member of the photosynthetic *Bradyrhizobium* [50]. However, bacterium AT1 did not form nodules on *Aeschynomene* plants and did not contain photosynthetic pigments [49]. The leaf, stem, and storage root extracts inoculated with AT1 showed nitrogenase (acetylene reduction) activity under microaerobic conditions (< 5% O_2_).

The genome of *Bradyrhizobium* sp. strain AT1 (MAFF107635) was composed of a single chromosome (7.5 Mb) with a *nif* gene cluster (*nifDKEN/nifS/nifB/nifHQV*) [51]. Although strain AT1 was phylogenetically close to *B. japonicum* USDA 6^T^ (a type strain of *B. japonicum*), the AT1 genome lacked symbiosis island and nodulation genes (*nodABC*), which are required for legume nodulation [51].

### 3.6. Diazotrophs Associated with Paddy Rice

The diazotrophic bacterial agents for plant growth promotion, isolated from the rhizosphere of rice plant, were *Agromonas oligotrophica* and *Burkholderia vietnamiensis* [52] and the agents isolated from the paddy rice tissues were *Aeschynomene*-nodulating *Bradyrhizobium* sp. [53,54], *Methylobacterium* sp. [55], and *Sphingomonas azotifigens* [56]. These endophytes, which colonized the rice plants, promoted the growth of the root and shoot masses accompanying the accumulation of phytohormones such as IAA and GA [57].

However, the isolation and inoculation of putative diazotrophic bacteria have not given definitive evidence for the existence of dominant endophytes that actively fix N_2_ within the rice tissues. Ueda et al. [58] conducted molecular analyses to identify active N_2_-fixing bacteria in rice roots collected from paddy fields in Japan. The search of 23 *nifH* gene sequences from rice root revealed γ-proteobacteria (*Klebsiella*, *Azotobacter*) and δ-proteobacteria (*Desulfovibrio gigas*) (Table 7). A metagenomic analysis of the nitrogen cycle, which functions inside the roots of field-grown rice plants, revealed the possible participation of *Bradyrhizobium* sp. strain BTAi1, *Xanthobacter autotrophicus*, and *Dickeya dadantii* for nitrogen fixation based on the detection of a fragment of *nifH* [59].

Organic matter degradation in submerged soil is accompanied by excessive production of methane (CH_4_) from the root exudates of photosynthates and exogenously added plant residues such as rice straws. When rice plants are grown in paddies, methane generated in rice rhizosphere is released into the atmosphere largely via plant aerenchyma [62]. The methane present in soil core surfaces and plant aerenchyma is actively oxidized by methanotrophs using the soil water-dissoluble oxygen and oxygen transported via the plant aerenchyma [63].

Certain methane-oxidizing bacteria (methanotrophs) are known to have N_2_-fixing *nifH* genes. The well-characterized N_2_-fixing methanotrophs are type II methanotrophs such as *Methylosinus* sp. and *Methylocystis* sp. [64]. Using the proteomic approach for the nitrogenase-derived peptides from paddy rice root tissues, Bao et al. [4] identified type II methanotrophs including *Methylosinus* sp. as the key players and *Methylocella, Bradyrhizobium, Rhodopseudomonas*, and *Burkholderia* as the minor players in N_2_ fixation. The relative abundances of these bacteria were increased in the paddy fields that received low-N fertilizer when compared to those that received high-N fertilizer [13]. Molecular analyses of the diazotrophs in paddy rice fields in Fujian, China [60], and IRRI (International Rice Research Institute), Philippines [61], indicated the participation of very diverse N_2_-fixing bacteria including methanotrophs and rhizobia (Table 7). Thus, significant biological N_2_ fixation by methanotrophs occurs using the biofuel of methane oxidation in addition to the common rhizosphere-associated N_2_ fixation in the paddy rice roots and rhizosphere [2].

## 4. Distribution and Ecophysiological Characteristics of Rhizobia in Non-Leguminous Plants

The molecular analyses of the distribution and N_2_-fixing activity of diazotrophs in the plant tissues (shoot, stems, roots) and root rhizosphere of six plant species (Table 1, Table 2, Table 3, Table 4, Table 5, Table 6 and Table 7) over the last 20 years has shown a high abundance of rhizobia, which are often capable of nodule formation and symbiotic N_2_ fixation in leguminous plants.

*Bradyrhizobium* spp., which may form stem/root nodules on *Aeschynomene* spp. and cowpea/siratro [42], were found in all six plant species of maize (Table 1), sorghum (Table 2), switchgrass (Table 3), sugarcane (Table 4 and Table 5), sweet potato (Table 6), and paddy rice (Table 7). *Bradyrhizobium* sp. ANU289, which may induce nodules on non-legume *Parasponia* sp. [65], was found in the stems of sweet potato (Table 6). *Azorhizobium* spp., which forms nodules on the stems and roots of *Sesbania rostrata*, has been found in sorghum (Table 2), Japanese sugarcane (Table 4), and sweet potato (Table 6). *Rhizobium* spp. has been found in maize (Table 1), switchgrass (Table 3), Japanese sugarcane (Table 4), Brazil sugarcane (Table 5), and sweet potato (Table 6).

*Burkholderia* spp. and *Ralstonia taiwanensis* of β-proteobacteria, which form root nodules on host plants of *Mimosa* [66,67,68], have been found in the tissues of switchgrass (Table 3), Japanese sugarcane (Table 4), Brazil sugarcane (Table 5), sweet potato (Table 6), and paddy rice (Table 7). *Methylobacterium nodulans*, which induces root nodules on *Crotalaria* and *Lotononis* legumes [69,70,71,72], was found in the roots of switchgrass, Japanese sugarcane (Table 4), and Brazil sugarcane (Table 5).

In the N_2_-fixing Japanese sugarcane (Table 4) and sweet potato (Table 6), a high expression of *nifH* RNA derived from presumed *Bradyrhizobium* spp. was detected. Expression of the nitrogenase peptides derived from *Bradyrhizobium* sp. BTAi1 was found in the roots of paddy rice (Table 7).

Here, we discuss three ecophysiological characteristics of these rhizobia that apparently constitute a symbiotic N_2_-fixing system in non-legumes without the formation of nodules. First, rhizobia often employ “crack entry” during the emergence of lateral roots on the primary and adventitious roots, and invade xylem parenchyma tissues via cortical cells [57], and more importantly, in cut sugarcane stem [10] and sweet potato tuber [48], diazotrophs exploit vegetative propagation for terminal colonization of the apoplasts rich in sugars, organic acids, and amino acids [73].

Molecular analyses of the wetland genus *Aeschynomene*, which nodulates *Bradyrhizobium* spp. (BTAi1, ORS278), indicated a lack of *nodABC* genes, which resulted in a Nod factor-independent infection process, likely via crack entry [74,75,76]. *B*. *oligotrophicum* S58^T^, isolated from the rice-paddy soil in Japan, had a genome similar to *Bradyrhizobium* spp. (BTAi1, ORS278) and lacked *nodABC* genes [77]. Indeed, *B*. *oligotrophicum* S58^T^ demonstrated good nodulation of *Aeschynomene indica,* similar to *Bradyrhizobium*. spp. (BTAi1, ORS278) [77].

Second, rhizobia often show free-living N_2_ fixation under microaerobic conditions. *Bradyrhizobium* spp., which nodulates *Aeschynomene* and *Parasponia*; *Azorhizobium caulinodans*, which nodulates *Sesbania rostrate*; and *Burkholderia*, which nodulates *Mimosa*, all have the ability to fix nitrogen in a free-living state (without host plants) under microaerobic conditions [65,67,78,79,80,81]. Endophytic N_2_ fixation by the rhizobia system [4] may take place in the microaerobic apoplasts located in intercellular tissues such as in sugarcane stem, which are rich in sugars (sucrose, maltose), organic acids (aconitate, succinate, malate, citrate), and amino acids [82,83,84].

More than 40 years ago, some *Rhizobium* species were shown to express nitrogenase activity in the free-living state, with or without combined N [85,86,87,88,89,90]. Specific sugars, organic acids, and N sources (glutamine/ammonia/nitrate) are a prerequisite to achieve high-N_2_-fixing activities (Figure 1C). In contrast to non-rhizobial bacteria such as *Klebsiella* sp., which assimilate fixed N totally for bacterial growth [91], a majority of fixed N in free-living rhizobial cells is exported as NH_4_^+^ [92,93]. The N_2_ fixation by rhizobia under microaerobic conditions is very active (as much as nodule bacteroids [93]), even in the excess of NH_4_^+^ [94], but assimilation of the fixed N (NH_4_^+^) into glutamine by glutamine synthetase is repressed in the presence of glutamine and NH_4_^+^ under microaerobic conditions [95]. The fixed N in the tissues of non-legumes may be excreted into the apoplast compartment under low pH (5.3–5.6) conditions [82,96]. The ammonia in the apoplasts may be taken up by plant cells for assimilation [97].

Third, rhizobia produce hormones to promote the growth of host plants in accordance with fixed N acquisition. Plant growth promotion, possibly by endophytic rhizobia, has frequently been reported. Yanni et al. [98] found that the growth of rice increased by 25–33% in the Egyptian rice paddies, where rice cultivation had been rotated with Egyptian berseem clover. This growth promotion cannot be explained solely by increased N supply from the mineralization of clover residues. The major attribute of the growth promotion may be due to the root-endophytic association of *Rhizobium leguminosarum* bv. trifolii, which produces plant hormones such as indole-3-acetic acid (auxin) and cytokinins [99]. The distribution of *R. leguminosarum* was also detected in the roots of sweet potato (Table 6).

Inoculation of photosynthetic *Bradyrhizobium* sp. strains, which colonize within rice roots, increased the shoot growth and grain yield of African wild rice *Oryza breviligulata* [53]. *Bradyrhizobium* spp. were found in the roots and stems of all six plant species studied (Table 1, Table 2, Table 3, Table 4, Table 5, Table 6 and Table 7). Many bacteria that have the ability to promote plant growth including *Burkholderia* spp. were also isolated from the roots and stems of plants. The selected bacteria can be used as biofertilizers in order to reduce the use of chemical N fertilizers [39].

## 5. Ecosystem Functioning of Diazotrophic Methanotrophs

Molecular analyses of the diazotrophs in the stems, roots, and rhizosphere indicated the distribution of methanotrophic bacteria. Type II methanotrophs (*Methylocella* sp., *Methylocystis* sp., *Methylosinus* sp.) were found in the stems of maize (Table 1); the roots of switchgrass (Table 3), Japanese sugarcane (Table 4), Brazil sugarcane (Table 5), and paddy rice (Table 7); and the rhizosphere of paddy rice (Table 7). These facts suggest the significance of N_2_ fixation by type II methanotroph across plant species other than paddy rice plants, although the expression is largely dependent on the CH_4_ supply in the environment (Figure 1D). The nitrogenase peptides derived from *Methylosinus* sp. and *Methylocystis* sp. were detected abundantly in the roots and rhizosphere of paddy rice (Table 7). *Beijerinckia* sp., which can also oxidize methane [100,101], was found in the roots of Japanese sugarcane (Table 4).

The methanotrophs generate reductant (NADH) and energy (ATP) through aerobic oxidation of environmental methane. The chemoautotrophic type II methanotrophs were found to exhibit N_2_-fixing ability using energy from the methane oxidation in the free-living state [64,102,103]. The reductants are supplied by the decarboxylation of methane-derived formic acid in the methane oxidation pathway [104,105]. However, further studies are required to understand the biochemistry of N_2_ fixation and the physiological processes of fixed-nitrogen utilization for plant growth and soil fertility in the methanotroph system.

Type II methanotrophs were found to be key players of N_2_ fixation in paddy rice roots [4,106]. This methanotrophic N_2_ fixation by rice paddies (around pH 6) may support the observed increase in soil N reserve by 4 kg ha^−1^ in soils growing rice, whereas the N reserves declined by 5 and 6 kg ha^−1^ in soils growing maize and wheat (without the methanotroph system), respectively [9].

Another hotspot for methanotrophic N_2_ fixation in the natural ecosystem is peatlands (around pH 4), where *Sphagnum* mosses are associated with acidophilic or acid-tolerant diazotrophic methanotrophs. A molecular analysis indicated that the methanotrophs in the peat bogs at the Mariapeel nature reserve, the Netherlands, were α-proteobacteria (*Methylocystis* sp. and *Methylosinus* sp.) and γ-proteobacteria (*Methylomonas* sp., *Methylosoma* sp., and *Methylovulum* sp.) [107], and methanotrophs in boreal peatlands in Alberta, Canada are *Methylocapsa acidiphila*, *Methyloferula stellata*, and *Methylocella palustris* (or silvestris) [108]. Thus, the *Sphagnum* moss methane-oxidizing diazotroph system serves as a methane filter, limiting methane emissions, and acting as an ecological N accumulator [107,109,110].

Although the acetylene reduction assay is generally used for the evaluation of N_2_ fixation, acetylene is a strong inhibitor of methane monooxygenase. Thus, this aspect of methane-dependent N_2_ fixation by methanotrophs has been ignored. To overcome this, ^15^N_2_ tracer experiments are required in non-leguminous plants such as paddy rice and mosses [103].

## 6. Research Perspectives

Our efforts to summarize studies on diazotrophs revealed that both rhizobia and methanotrophs play important roles in N_2_ fixation in non-leguminous plants (Figure 1). Molecular analyses of transcription and translation of *nifHDK* genes encoding nitrogenase have been a powerful approach to identify functional N_2_-fixing bacteria in non-leguminous plants, which are able to adopt other plants for the identification of functional diazotrophs. However, several questions need to be solved for N_2_ fixation and associations of rhizobia and methanotrophs in non-leguminous plants.

Data on *nif* gene expression and nitrogenase activity in non-legume diazotrophs have provided a snapshot of different plant contexts such as plant growth stage [20] and circadian rhythm [111] under fluctuations of carbon supply from plants. Time series analysis based on the development of sequence technologies [112,113,114] would reveal the dynamics of truly functional diazotrophs in plant tissues and rhizosphere.

Other questions on the mechanisms by which rhizobia and methanotrophs associate with plant tissues and microbial communities affect the distribution and functions of diazotrophs in non-leguminous plants still exist. Recent studies on plant microbiome have indicated that the majority of bacterial species within the Rhizobiales order are consistently enriched in the roots and shoots of plants [13,61,115]. Genome comparisons of the Rhizobiales members including rhizobia have suggested that they originally possessed multiple genes for gibberellin biosynthesis, chemotaxis, and type III VI protein secretion systems that have helped them to adapt to plant environments [115,116]. The ancestor of Rhizobiales was fully adapted to plant environments, and its lineages have acquired genes for nitrogen fixation and nodulation [115,116]. In this regard, diazotrophs of type II methanotrophs such as *Methylocystis* and *Methylosinus* are members of the *Rhizobiales*, suggesting their potential for adaptation to root endophytic environments [106].

Taken together, the primary focus of research on N_2_-fixing bacteria in plants is to understand the functioning of plant microbiomes: how microbial communities regulate the growth, health, and productivity of plants [113,117]. Future genomics and functional studies on rhizobia and methanotrophs from field-grown non-leguminous plants would provide a better understanding of their lifestyle and strategies to adapt to diverse plant environments, and thereby help us to maximize their beneficial functions in different agricultural settings.

## Figures and Tables

**Table 1:** Detection of *nifH* genes in the tissues and rhizosphere soil of field-grown maize plants.

Site and Sample of Investigation	Detection of *nifH* Genes	Close Genus Abundance and Bacteria	
Stem harvested in six regions in Rio Grande do Sul, Brazil [17]	Sequencing of *nifH* DNA clones	27% 20% 12.6% 7.4% 5.4% The others restricted to the stem	*Ideonella* *Azospirillum* *Klebsiella* *Herbaspirillum* *Raoultella* *Methylosinus*, *Rhizobium*
Root harvested in six regions in Rio Grande do Sul, Brazil [17]	Sequencing of *nifH* DNA clones	30% 23.3% 11.9% The other restricted to the root	*Bradyrhizobium* *Azospirillum* *Klebsiella* *Dechloromonas*
Rhizosphere soil collected in six regions in Rio Grande do Sul, Brazil [17]	Sequencing of *nifH* DNA clones	20.8% 11% 5% 0.2% The others restricted to the rhizosphere soil	*Bradyrhizobium* *Ideonella* *Azospirillum* *Klebsiella* *Methylocystis*, *Beijerinckia*, *Geobacter*, *Rhodovulum*, *Methylobacterium*, *Gluconacetobacter*, *Methylocella*, *Delftia*

**Table 2:** Detection of *nifHDK* genes in the tissues and rhizosphere soil of field-grown sorghum plants.

Site and Sample of Investigation	Detection of *nifHDK* Genes and Their Proteins	Close Genus (Similarity >91%) Abundance and Bacteria	
Roots harvested from two sorghum lines (KM1, KM2) at late growth stage in a Fukushima field, Japan [20]	Metagenome for *nifHDK* genes	68% (KM1), 88% (KM2) 1–3% (KM1, KM2)	*Bradyrhizobium* spp. (including *B.* sp. S23321 and *B. oligotrophicum* S58^T^) *Azorhizobium* sp.
	Proteome for NifHDK proteins	71% (KM1), 69% (KM2)	*Bradyrhizobium* spp. (including *B.* sp. S23321 and *B.* sp. S58^T^)
Rhizosphere of two cultivars (IPA 1011, IS 5322-C) with low (LF) and high fertilizer (HF) in Cerrado soil, Brazil [19]	Sequencing of *nifH* DNA clones	In IPA-LF 43% 21% 7% 18% In IPA-HF 29% 10% 16% 7% 8% 5% In IS-LF 42% 10% 10% 13% In IS-HF 39% 23% 11% 10% 13%	*Bradyrhizobium* sp. *B.* sp. AF484629 *Rhizobium etli* *Azohydromonas australica* *Bradyrhizobium* sp. *B.* sp. AF484629 *Rhizobium etli* *Azohydromonas australica* *Ideonella* sp. *Burkholderia vietnamiensis* *Bradyrhizobium* sp. *B.* sp. AF484629 *Azohydromonas australica* *Ideonella* sp. *Bradyrhizobium* sp. *B.* sp. AF484629 *Sinorhizobium* sp. *Azohydromonas australica* *Ideonella* sp.

**Table 3:** Detection of *nifH* DNA and *nifH* RNA in the tissues of field-grown switchgrass plants.

Site and Sample of Investigation	Detection of *nifH* Genes	Close Genus Abundance and Bacteria	
Shoots from the tallgrass prairie of northern Oklahoma, USA [21]	Sequencing of *nifH* DNA clones	7% 19% 6% 15% 11% 12% 6% 5% 4% 4% 4%	*Bradyrhizobium* sp. BTAi1 *B.* sp. MAFF210318 *Burkholderia* spp. *Sphingomonas azotifigens* *Rhizobium helanshanense* *Desulfuromonas* spp. *Azospirillum lipoferum* *Klebsiella* sp. *Anaeromyxobacter* spp. *Geobacter* spp. *Syntrophobacter fumaroxidans*
Roots from the tallgrass prairie of northern Oklahoma, USA [21]	Sequencing of *nifH* DNA clones	9% 12% 18% 17% 21% 13% 14% 6% 1.5%	*Bradyrhizobium* sp. BTAi1 *B.* sp. MAFF210318 *B. japonicum* *Burkholderia* spp. *Sphingomonas azotifigens* *Anaeromyxobacter* spp. *Geobacter* spp. *Methylocystis* sp. *Methylobacterium nodulans*
	RT-PCR amplification of *nifH* RNA	10% 36% 13% 7% 9% 15%	*Burkholderia* spp. *Rhizobium helanshanense* *Desulfuromonas* spp. *Geobacter* spp. *Azoarcus* sp. BH72 *Methylobacterium nodulans*

**Table 4:** Detection of *nifH* genes and their expression in the tissues of sugarcane plants grown in Japan.

Site and Sample of Investigation	Detection of *nifH* Genes	Close Genus Abundance and Bacteria	
Stems of 8-month-old sugarcane cv. KF92-93, cv. NCo310 and cv. NiF8 grown in Miyako Island, Japan [11]	Sequencing of *nifH* DNA clones	100% (KF), 88% (NCo) 73% (NiF) 27% (NiF)	*Bradyrhizobium* spp. *Klebsiella* spp. *Serratia* spp.
Stems of 50- and 100-day-old sugarcane (cv. NiF8) grown on a commercial soil under high temperature [10]	Sequencing of *nifH* DNA clones	22% (50), 19% (100) 17% (50), 16% (100) 19% (50), 19% (100) 15% (50), 13% (100) 22% (50), 13% (100)	*Bradyrhizobium* sp. BTAi1 *B.* sp. IRBG230 *B.* sp. MAFF210318 *Azorhizobium caulinodans* *Rhizobium daejonense*
	RT-PCR amplification of *nifH* RNA	87% (100) 5% (50), 4% (100) 52% (50)	*B* sp. IRBG230 *B.* sp. MAFF210318 *Azorhizobium caulinodans*
Roots of 50- and 100-day-old sugarcane (cv. NiF8) grown on a commercial soil under high temperature [10]	Sequencing of *nifH* DNA clones	30% (50), 32% (100) 25% (50), 25% (100) 12% (50), 13% (100) 7% (50), 9% (100) 23% (50), 22% (100)	*B.* sp. MAFF210318 *B.* sp. IRBG230, *Azorhizobium caulinodans* *Rhizobium daejonense* *Beijerinckia derxii*
	RT-PCR amplification of *nifH* RNA	19% (50) 100% (100) 5% (50) 17% (50) 23% (50)	*B.* sp. MAFF210318 *B.* sp. IRBG230, *Azorhizobium caulinodans* *Sinorhizobium fredii* *Beijerinckia derxii*
Roots of 59- and 100-day-old sugarcane (cv. NiF8) grown on Ishigaki soil under low temperature [10]	Sequencing of *nifH* DNA clones	14% (59), 19% (100) 20% (59), 19% (100) 39% (59), 37% (100) 6% (59), 7% (100) 14% (100)	*B.* sp. MAFF210318 *B.* sp. IRBG230, *Rhizobium daejonense* *Methylocystis rosea* *Methylobacterium* sp.
	RT-PCR amplification of *nifH* RNA	50% (59), 100%(100) 50% (59)	*B.* sp. BTAi1 *Burkholderia ferrariae*
Roots of 59- and 100-day-old sugarcane (cv. NiF8) grown on Tanegashima soil under low temperature [10]	Sequencing of *nifH* DNA clones	46% (59), 24% (100) 18% (59), 29% (100) 4% (59), 15% (100) 12% (100) 4% (59) 6% (59)	*B.* sp. MAFF210318 *B.* sp. IRBG230, *B.* sp. IRBG228, *Methylobacterium nodulans* *Methylocella silvestris* *Azonexus caeni*
	RT-PCR amplification of *nifH* RNA	100% (100)	*B.* sp. MAFF210318

**Table 5:** Detection of *nifH* genes and their expression in the tissues of sugarcane plants grown in Brazil.

Site and Sample of Investigation	Detection of *nifH* Genes	Close Genus Abundance and Bacteria	
Leaf sheath of 6-month-old sugarcane (cv. RB 867515) grown in EMBRAPA without fertilizer and inoculation [41]	Determination of 16S rRNA cDNA sequences	81% 19%	α*-Proteobacteria* (mostly *Gluconacetobacter*) β-*Proteobacteria* (*Burkholderia* spp., *Herbaspirillum* spp.)
	RT-PCR amplification of *nifH* RNA	10% 6% 84%	*Rhizobium* spp. *Paraburkholderia tropica* *Idenella/Herbaspirillum*-like bacteria
Root of 6-month-old sugarcane (cv. RB 867515) grown in EMBRAPA without fertilizer and inoculation [41]	Determination of 16S rRNA cDNA sequences	42% 3% 11% 17% 2% 25%	α*-Proteobacteria* (*Rhizobium* spp., *Bradyrhizobium* spp.) β*-Proteobacteria* δ*-Proteobacteria* *Actinobacteria* *Acidobacteria, Planctomycetes*
	RT-PCR amplification of *nifH* RNA	8% 20% 36% 24% 12%	*Azospirillum brasilense* *Bradyrhizobium* spp. *Methylocapsa* spp. *Paraburkholderia tropica* *Idenella/Herbaspirillum*-like
White shoot roots of 5-month-old sugarcane (cv. RB867515) grown on EMBRAPA field [42]	Trap-plant (siratro) isolates Determination of 16S rRNA and cDNA sequence	96% 4%	*Bradyrhizobium* spp. *Rhizobium* sp. (no *nodC*)
	Trap-plant (cowpea) isolates Determination of 16S rRNA and cDNA sequence	23% 3%	*Bradyrhizobium* spp. *Rhizobium* spp.
	Direct plate isolates	6/9 1/9 1/9 1/9	*Bradyrhizobium* spp. (4 no *nodC*) *Rhizobium* sp. (no *nodC*) *Methylobacterium* *Herbaspirillum*

**Table 6:** Detection of *nifH* genes in the tissues of field-grown sweet potato plants.

Site and Sampling of Investigation	Detection of *nifH* Genes	Close Genus (Similarity >91%) Abundance and Bacteria	
Stem of African sweet potato grown in Uganda and Kenya [47]	Sequencing of *nifH* DNA clones	17% (Kenya) 71% (Kenya) 83% (Uganda)	*Bradyrhizobium* sp. ANU 289 *Azoarcus* sp. BH72 *Clostridium pasteurianum*
Stem harvested in Oct. 2002, Aug. 2004 and Oct. 2004 from cv. Beniazuma grown in Andozol, Japan [48]	Sequencing of *nifH* DNA clones	31% (O2) 18% (O4) 18% (O4) 100% (A4), 63% (O4)	*Herbaspirillum seropedicae* *B.* sp. MAFF210318 *B.* sp. IRBG230 *Azohydromonas australica*
	PCR amplification of *nifH* RNA	77% (O2) 22% (O2) 40% (A4) 60% (A4)	*Bacillus* sp. BT97 *B.* sp. IRBG228 *B.* sp. MAFF210318 *B.* sp. IRBG230
Stem harvested in Oct. 2005 and Aug. 2006 from cv. Ayamurasaki grown on a gray lowland soil, Japan [48]	Sequencing of *nifH* DNA clones	100% (O5) 27% (A6) 18% (A6) 18% (A6) 36% (A6)	*B.* sp. IRBG230 *Pelomonas saccharophila* *Azohydromonas australica* *Paraburkholderia unamae* *Tolypothrix* sp. PCC7601
	PCR amplification of *nifH* RNA	100% (A6)	*Pelomonas saccharophila*
Tuber of African sweet potato grown in Uganda and Kenya [48]	Sequencing of *nifH* DNA clones	28% (Kenya) 71% (Uganda), 14% (Kenya) 71% (Kenya) 29% (Uganda), 28% (Kenya)	*Bradyrhizobium japonicum* *Sinorhizobium meliloti* *Azoarcus* sp. BH72 *Paenibacillus odorifer*
Tuber harvested in Oct. 2002, and Oct. 2004 from cv. Beniazuma grown on an Andozol, Japan [48]	Sequencing of *nifH* DNA clones	46% (O2) 23% (O2), 30% (O4) 70% (O4)	*B.* sp. MAFF210318 *Bradyrhizobium japonicum* *Rhizobium leguminosarum*
	PCR amplification of *nifH* RNA	15% (O2) 85% (O2)	*Bradyrhizobium japonicum* *Bacillus* sp. BT97
Tuber harvested in Oct. 2005, Aug. 2006, and Oct. 2006 from cv. Ayamurasaki grown on a gray lowland soil, Japan [48]	Sequencing of *nifH* DNA clones	46% (O5) 25% (A6), 28% (O6) 14% (O6) 10% (O5), 33% (A6), 21% (O6) 13% (O5), 17% (A6), 14% (O6) 18% (O5)	*Azorhizobium caulinodans* *B.* sp. IRBG230 *Sinorhizobium* sp. *Pelomonas saccharophila* *Azohydromonas australica* *Paraburkholderia vietnamiensis*
	PCR amplification of *nifH* RNA	100% (A6)	*B.* sp. IRBG230

**Table 7:** Detection of *nifHDK* genes and their expressed proteins in the tissues and rhizosphere of field-grown paddy rice.

Site and Sample of Investigation	Detection of *nifHDK* Genes and Their Proteins	Closest Genus Abundance and Bacteria	
Roots harvested from paddy rice grown on Kyushu University field [58]	Sequencing of *nifH* DNA clones		γ-Proteobacteria (*Klebsiella pneumoniae, Azotobacter*) δ-Proteobacteria (*Desulfovibrio gigas*)
Roots harvested from paddy rice (cv. IR55423-01) grown in IRRI field, the Philippines at flowering [59]	Metagenome for *nifH* DNA	3/5 1/5 1/5	*Bradyrhizobium* sp. BTAi1 *Xanthobacter autotrophicus* *Dickeya dadantii*
	RT-PCR amplification of *nifH* RNA		*Geobacter* spp.
Roots harvested from cv. Nipponbare rice grown on Tohoku University field at flowering stage [4]	Metaproteome for NifHDK	29.7% 21.8% 9.3%	*Methylocystaceae* (*Methylosinus* sp., *Methylocystis* sp.) *Bradyrhizobiaceae* (*Bradyrhizobium*, *Rhodopseudomonas*) *Burkholderiaceae*
Rhizosphere from paddy rice field of Fujian province, China [60]	RT-PCR amplification of *nifH* RNA	4 clones 3 clones 4 clones	α-Proteobacteria (*Rhizobium, Methylocystis*) β-Proteobacteria (*Azoarcus* sp., *Azospira oryzae*, *Azotobacter* sp.) γ-Proteobacteria (*Methylococcus*) δ-Proteobacteria (*Geobacter*) Firmicutes (*Helicobacter*)
Rhizosphere collected at IRRI fields, the Philippines 59 to 76 days after rice transplanting [61]	Metagenome 16S rRNA		α-Proteobacteria (*Rhizobium, Methylobacterium*) Actinobacteria (*Microbacterium*)
	Sequencing of *nifH* DNA clones		*Rhizobium, Methylococcus, Dechloromonas, Anaeromyxobacter, Syntrophobacter,* some methanogenic archaea
	Metaproteome	33%	α-Proteobacteria (*Bradyrhizobium, Rhodopseudomonas, Azospirillum, Methylobacterium, Magnetospirillum, Methylosinus*) β-Proteobacteria (*Dechloromonas, Acidovorax, Herbaspirillum*) δ-Proteobacteria (*Anaeromyxobacter, Geobacter, Desulfovibrio*)
